# AFLP-based genetic diversity of wild orchardgrass germplasm collections from Central Asia and Western China, and the relation to environmental factors

**DOI:** 10.1371/journal.pone.0195273

**Published:** 2018-04-11

**Authors:** Chenglin Zhang, Ming Sun, Xinquan Zhang, Shiyong Chen, Gang Nie, Yan Peng, Linkai Huang, Xiao Ma

**Affiliations:** 1 Department of Grassland Science, Animal Science and Technology College, Sichuan Agricultural University, Chengdu, China; 2 College of Life Science and Technology, Southwest University for Nationalities, Chengdu, China; Brigham Young University, UNITED STATES

## Abstract

*Dactylis glomerata* L. (orchardgrass) is an important perennial forage species in temperate areas of the world. It is usually used for silage, grazing and hay because of its high nutritional value and reproducibility. Central Asia, Xinjiang and Tibetan Plateau in China possess various special micro-environments that harbor many valuable resources, while different degrees of degradation of the grassland ecosystem occurred due to climatic changing and human activities. Investigating the genetic diversity of wild *D*. *glomerat* could provide basis for collection, protection, and utilization of some excellent germplasm resources. Totally 210 individuals from 14 populations—five from Xinjiang, two from Kangding (Tibetan Plateau), and seven from Central Asia were identified using AFLP technology. The average values of Nei’s genetic diversity (*H*_*j*_) and Shannon information index (*H*_*o*_) were 0.383 and 0.394 respectively. UPGMA tree, STRUCTURE analysis and principal coordinate analysis (PCoA) showed populations from same region clustered together. AMOVA revealed 35.10% of the genetic differentiation (*F*_*st*_) occurred among populations. Gene flow (*N*_*m*_) was limited among all populations. Genetic diversity of *D*. *glomerata* was high but limited under isolation-by-distance pattern, resulting in high genetic differentiation and low gene flow among populations. Adjacent regions also exhibited similar results because of the barriers of high mountains. The environmental factors, such as precipitation, elevation, latitude and longitude also had some impacts on genetic diversity and structure pattern of populations.

## Introduction

*Dactylis glomerata* L., a perennial cool-season forage grass, belongs to the monotypic genera *Dactylis*, known as orchardgrass or cocksfoot [1]. The basic chromosome number of *Dactylis* is 2n = 14 and, although the species consists of diploid, tetraploid and hexaploid, the commonly used forage form is tetraploid [2,3]. With agronomically and economically importance, *Dactylis* is the fourth widely used grass genus following *Lolium*, *Festuca and Phleum* in global temperate areas, used as silage, hay or grazing [4–8]. *D*. *glomerata* is a self-incompatible, wind-pollinated, outcrossing species, therefore various levels of ploidy and high phenotypic variation existed among species during the long term natural selection and adaptation processes [6,9]. The nuclear internal transcribed spacer (ITS) research and trnL intron chloroplast sequences have revealed that *Dactylis* very likely originated as *D*. *glomerata* ssp. *altaica* in Central Asia [10]. During the migration to Europe and East Asia, species ploidy partly increased and formed the current cosmopolitan distribution of the subspecies. The diploids mainly grow in low-density forest-floor habitat in woodlands, whereas the tetraploids widely distribute in varied habitats in open areas, especially in disturbed anthropic sites [11,12].

*D*. *glomerata* is an important and valuable model grass to investigate the genetic diversity due to its agricultural importance, wide distributed limits, and resistance to diverse stress [13,14]. Such investigations are essential to understand the biological processes within and among populations during historic events (e.g., founder effects, population bottlenecks, habitat fragmentation, etc.), and directly indicate the impacts of environment on individual genotypes [15,16]. Generally, polyploid populations with a broad geographically distributed range are better suited to survive and colonize new niches in diverse environments, because they occupy greater heterozygosity and variation, lower inbreeding depression [4,17]. The specific environmental conditions, such as elevation, latitude, precipitation, sunlight may lead to different physiological challenges for species, which in turn results in morphological and molecular adaptation to local environment [18]. So the genomes of populations in different environments may genetically differ at a few key sites and contain valuable alleles. Thus, assessing the level of genetic diversity among different geographical regions benefits for forage crop breeding, germplasm collection, and protection of genotypes from genetic erosion [19–21].

As one of the original centers of *Dactylis*, various environmental conditions in Central Asia possess many valuable alleles or traits of species. These complex micro-environments are derived by mountain orogeny, which has been associated with some diversification and speciation of plants [22]. For instance, the Tianshan Mountains in Xinjiang of China and Central Asia, are comprised by three parallel east-west mountain chains. They are formed by historical glaciation and orogenic movement, and seriously affect species diversity since Holocene epoch. These latitudinally- trending mountain chains act as an East–West passway allowing species lineage exchange, but a North–South barrier promoting vicariance and genetic differentiation due to the large-scale glacial cap on mountaintop [23].

The south Tibetan Plateau is one of the native distributed areas of *Dactylis* in China. Tibetan Plateau now is a still uplifted geographical region that is encircled by the western Karakorum massif, the northern Kunlun mountains, the southern Himalayas and deeply incised mountain ranges [24,25]. It is the world’s highest rangeland landscapes that harbor many kinds of cultural and biological resources. Its alpine ecosystems contain many vegetation types such as semiarid steppe, shrub lands, alpine steppe, moist alpine meadows and so on, which support a rich array of unique floral and faunal assemblages [26,27]. While the increased livestock numbers and climatic changing have resulted in degradation of the grassland ecosystem in these regions, causing genetic erosion and seriously influencing the ecological security and development of animal husbandry in local regions [28]. So more diverse investigations and protection programs for native species in Tibetan Plateau is necessary and valuable.

Traditional evaluation by phenotypic traits takes much time and endeavor to rebuild the homogenous environment for individuals in replicated field experiments [29]. Molecular markers are more effective and reliable to evaluate germplasm resources. Amplified fragment-length polymorphisms (AFLP) is a robust and highly informative DNA fingerprinting technique that provides more detailed information about genetic variation and diversity on DNA-level, because it could deal with a large number of polymorphic loci without prior information of genomes [30,31]. Therefore it has been widely used for investigation of germplasm genetic diversity detection, high density linkage map construction, gene localization and cloning, and identification of cultivars [32]. The previous researches of *D*. *glomerata* mostly adopted morphological method or bulked DNA strategy, while the investigations on population genetic diversity that using individual DNAs rarely report yet [3,4,8,33]. Marking on individual DNA is more effective to distinguish geographically distinct populations, which could differ in the level of genetic diversity or in the distribution of diversity within and among regions.

The primary focus of most previous investigations is on contemporary phenotypes and diversity of current populations, but the potential driving force of specific selective evolution in population divergence is often not addressed [34]. Generally, neutral evolution and natural selection partly have the joint effects on population divergence and speciation [35]. When gene flow is broken off, divergence and differentiation among populations would intensify if random drift driven, and divergent selection might lead to local adaptation [35,36]. However, it is difficult to determine genetic mechanism of the environmental effects on plant phenotypes ignoring the outlier loci that under specific selection pressures. Identifying outlier loci shows signatures of natural selection and implicitly demonstrates non-outlier loci under neutrally evolution between populations. So studying the population structure using outlier loci is an important step to address alternate explanations for population differentiation under neutral evolution and/or natural selection, especially for distinctly structured populations [37].

Totally 14 wild *D*. *glomerata* populations from three geographical regions were used to research their genetic diversity and structure patterns, and develop basis for the collection, protection, and utilization of some excellent germplasm resources. Accordingly, the objectives were: (1) to estimate genetic diversity and structure pattern of populations, (2) to study the gene flow (vs. genetic drift) among regions, (3) to evaluate the correlation of genetic diversity and environmental factors.

## Materials and methods

### Plant materials and DNA extraction

**Fig 1:**
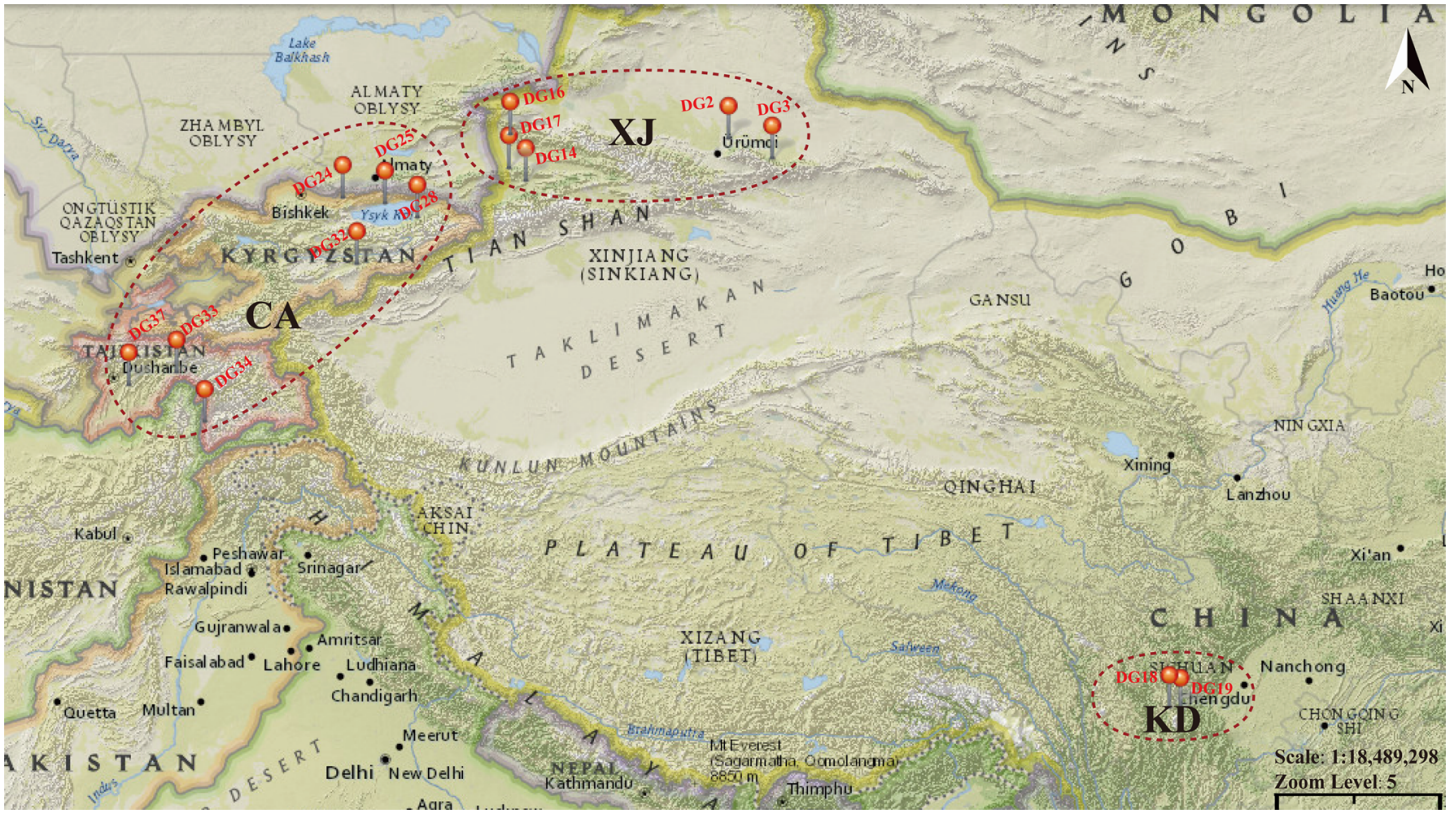
Geographical locations of analyzed populations of *Dactylis glomerata*.

Fourteen wildland site collections of *D*. *glomerata* were used (Fig 1), with 15 plants per collection (S1 Table), for a total of 210 individuals. These wild populations were collected by NPGS genebank of USDA from Central Asia (CA), Xinjiang (XJ) and Kangding (KD) of China (https://npgsweb.ars-grin.gov/gringlobal/taxonomydetail.aspx?id=13114), and had not been cultivated or actively managed for pasture improvement. Seed was collected from fifty or more randomly sampled individual plants in each population. The sampling sites ranged from the northernmost population DG2 at Heavenly Lake in Xinjiang (44°7′0″N) to the southernmost population DG19 in Kangding (29°59′0″N), and from the easternmost population DG19 in Kangding (101°57′25″E) to the westernmost population DG37 in Kulyab of Tajikistan (69°14′29″E). Some other climate factors (*i*.*e*., altitude, annual mean temperature, annual precipitation) of each sampling site were obtained from DIVA-GIS software [38]. S1 Table presented origins, climatic information and habitat for 14 populations. Seeds were planted in containers in a phytotron at Sichuan Agriculture University (Chengdu, China). Before DNA extraction, identification of chromosome number was carried out for each population by squashed root tips technique, and the results were displayed in S1 Table.

Each individual sample was comprised by 4 ~ 5 fresh leaf tissues (about 70 mg), and genomic DNA was extracted using plant DNA extraction kits (Tiangen, Beijing, China). NanoDrop^®^ ND-1000 Spectrophotometer (NanoDrop Technologies, USA) was used for quantifying the DNA concentration.

#### AFLP procedure

AFLP reactions were performed following Sun et al. [2] with a few minor modifications. Six informative and reliable pairs of *Mse*I (M-GACGATGAGTCCTGAG) and *Eco*RI (E-CTCGTAGACTGCGTACC) primers used in amplification were selected from a set of 100 primer pairs. The amplification PCR reaction was performed in a final volume of 20 μL as Zhang et al. [39]. The capillary electrophoresis was performed using ABI 3500 DNA analyzer (Applied Biosystems, USA) and those fragments were analyzed using GeneMarker^®^ (version 2.6, SoftGenetics, LLC).

### Data analysis

#### Band statistics

Each band of AFLP amplification was treated as a separate locus. The clear bands were recorded as presence (1) and the alternative were absence (0) ranging from 80 and 400 bp to generate the binary matrices for further analyses.

#### Outlier detection

In order to identify candidate AFLP loci that potentially influenced by selection, two kinds of approaches were performed: DetSel v1.02 [40], a free package in R program [41] was used to identify outlier loci with Bayesian likelihood method under MCMC (Monte Carlo Markov chain). The burn-in of 50,000 iterations and sample size of 10,000 were used under a thinning interval of 50. Typically, population-specific and locus-specific genetic differentiation (*F*_*st*_) is used and allele frequencies are Dirichlet distributed in the hypothesis of Bayesian methods [42]. Previous logarithmic criterions have considered that the locus is an outlier as the threshold of PO > 10 (log_10_ PO > 1.0) [43,44]. For the second approach, Mcheza program was used to detect the signatures of natural selection [45]. It calculated the mean *F*_*st*_ forcing the simulation values for heterozygosity (*H*_*e*_) with 1,000,000 Markov chain simulations. Totally three times were conducted at 95% confidence interval in order to ensure the accuracy of converged inference.

#### Genetic diversity

Linkage disequilibrium (LD) was calculated for each population using ARLEQUIN v2.0 [46] to detect recent population bottlenecks and exaction-replacement [47] (at α = 0.05). At primer level, the total number of bands (TNB), the number of polymorphic bands (NPB), the percentage of polymorphic bands (PPB) and the polymorphism information content (PIC) [48] were calculated by using Excel 2013 software. Shannon information index (*H*_*o*_) was calculated using POPGENE v3.0 [48], assuming populations under Hardy-Weinberg equilibrium (HWE). At species level, NPB, PPB and *H*_*o*_ were calculated. POPGENE v3.0 [48] was used to compute the observed number of alleles per locus (*N*_*a*_) and the effective number of alleles per locus (*N*_*e*_). And the Nei’s genetic diversity (*H*_*j*_) was computed using AFLP-SURV v1.0 [2].

#### Genetic structure

To analysis the genetic structure, we used the UPGMA (unweighted pair group method with arithmetic mean) clustering procedure for the 14 populations of *D*. *glomerata*. We firstly computed Nei’s genetic distance (GD) matrix with 10,000 bootstrap values using AFLP-SURV v1.0 [2]. Then the GD matrix was used for computing the dendrogram by the CONSENSE module in PHYLIP v3.69 [49]. The principal coordinate analysis (PCoA) of 210 individuals was carried out in “stats” [41] and “scatterplot3d” [50] in R program for the visualization of their spatial structure. We also used Bayesian model-based cluster analysis using STRUCTURE v2.3.4 [51] to infer genetic structure and to define the number of clusters in the data set. The correlated allele frequencies and admixed model were applied with 50,000 burn-in and 100,000 MCMC. The assumed clusters (K) were from 1 to 14 and 10 runs were carried out for each K. The STRUCTURE HARVESTER [52] was used for the determination of optimum K based on L(K) or ΔK, which were usually plateaued after the “optimum K” was reached. For further assessing the genetic distinctiveness of populations, an assignment test was computed to reallocate individuals back to their original populations using AFLPOP v1.1 [53]. The resampling of 1,000 artificial genotypes under the minimum log-likelihood difference of 2 was conducted: replacing zero frequencies by (1 / (sample size + 1)) and calculating the p-value of log-likelihood for each individual. The individual wouldn’t be allocated to any of the candidate populations as its p-value was below a certain threshold (< 0.001).

#### Genetic differentiation

To study the magnitude of genetic differentiation (*F*_*st*_), the partitioning of variation at different scale was computed with Analysis of Molecular Variance (AMOVA) in “vegan” [54] in R program. Shannon differentiation coefficient (*G’*_*st*_) was also estimated as *G’*_*st*_ = (*H*_*sp*_ − *H*_*pop*_) / *H*_*sp*_ (*H*_*sp*_: total Shannon information index, *H*_*pop*_: average Shannon information index within populations). The gene flow (*N*_*m*_) among populations was conducted as *N*_*m*_ = (1 − *F*_*st*_) / 4 *F*_*st*_.

#### Correlation analysis

Mantel test was performed between pairwise *F*_*st*_ and geographical distance for detecting the isolation-by-distance (IBD) [55] in “ade4” [56] in R program. The regression analyses between *H*_*j*_ and environmental factors (average temperature, annual precipitation and altitude) were estimated using R package “stats” [57] with 99% confidence intervals.

## Results

### AFLP and potentially adaptive loci

Totally 643 clear bands were generated and 352 of them were polymorphic using 6 AFLP primer pairs (See S2 Table with the amplified information of 6 primer pairs). Each primer pair produced an average of 107.2 distinct bands with an average of 58.7 polymorphic bands. The polymorphic rate ranged from 50.50% to 60.19% with an average of 54.73% and the polymorphic information content (PIC) of each primer pairs ranged from 0.169 to 0.198 with an average of 0.179. The Shannon information index (*H*_*o*_) of each pair of primers ranged from 0.2945 to 0.3097 with an average level of 0.3026.

Among the 352 loci, a total 32 of them (9.09%) significantly took higher values of PO >10 in DetSel (See S1 Fig with this article). In Mcheza program, the *F*_*st*_ values of 38 loci (10.80%) were significantly high deviating from 95% confidence intervals (See S2 Fig with this article). The two detection methods totally revealed 22 same potential outliers that were considered candidate loci under divergent selection.

### Population genetic diversity

Linkage disequilibrium (LD), or non-random association of alleles was calculated for each population at the 5% level, revealing a low proportion of the polymorphic loci pairs (8.40%, *p* < 0.05) (Table 1). For genetic diversity at the population level, the number of polymorphic loci ranged from *N*_*p*_ = 137 by population DG18 to *N*_*p*_ = 233 by population DG28. Population DG24 showed a slightly higher genetic diversity (*N*_*e*_ = 1.33, *H*_*j*_ = 0.228, *H*_*o*_ = 0.294) relative to population DG32 (*N*_*e*_ = 1.32, *H*_*j*_ = 0.219, *H*_*o*_ = 0.289), and population DG18 presented the lowest level (*N*_*e*_ = 1.24, *H*_*j*_ = 0.163, *H*_*o*_ = 0.201). At the regional level, the genetic diversity was lowest in Kangding (*N*_*e*_ = 1.27, *H*_*j*_ = 0.274, *H*_*o*_ = 0.232) and highest in Central Asia (*N*_*e*_ = 1.36, *H*_*j*_ = 0.225, *H*_*o*_ = 0.352). The total genetic diversity of the species was high (*N*_*e*_ = 1.39, *H*_*j*_ = 0.233, *H*_*o*_ = 0.394).

**Table 1:** Genetic diversity of *D*. *glomerata* distributed among populations in different sampling regions. Codes: LD, linkage disequilibrium at the 5% level; *N*_*p*_, Number of polymorphic loci; PPL, percentage of polymorphic loci; *N*_*a*_, observed number of alleles per locus; *N*_*e*_, effective number of alleles per locus; *H*_*j*_, Nei’s gene diversity index; *H*_*o*_, Shannon information index.

Population	LD (%)	*N*_*p*_	PPL (%)	*N*_*a*_	*N*_*e*_	*H*_*j*_	*H*_*o*_
DG2	9.19	178	50.57	1.51	1.26	0.283±0.039	0.236±0.070
DG3	8.56	189	53.69	1.54	1.28	0.253±0.039	0.251±0.070
DG14	9.44	231	65.62	1.66	1.28	0.284±0.037	0.273±0.066
DG16	9.12	221	62.78	1.63	1.26	0.258±0.036	0.255±0.065
DG17	7.18	197	55.97	1.56	1.31	0.293±0.041	0.275±0.073
DG18	9.01	137	38.92	1.39	1.24	0.243±0.040	0.201±0.072
DG19	9.14	147	41.76	1.42	1.25	0.256±0.040	0.216±0.072
DG24	5.74	216	61.36	1.61	1.33	0.308±0.039	0.294±0.071
DG25	8.58	229	65.06	1.65	1.30	0.295±0.038	0.286±0.067
DG28	9.34	233	66.19	1.66	1.31	0.274±0.038	0.288±0.068
DG32	9.91	219	66.22	1.62	1.32	0.262±0.040	0.289±0.071
DG33	7.03	200	56.82	1.57	1.28	0.248±0.040	0.257±0.070
DG34	6.14	213	60.51	1.61	1.29	0.266±0.039	0.269±0.069
DG37	9.18	205	58.24	1.58	1.30	0.291±0.040	0.270±0.071
Total	8.40	352	100	2.00	1.39	0.383±0.152	0.394±0.013
Mean	8.40	201.07	57.41	1.57	1.29	0.272±0.089	0.261±0.023

### Population genetic structure

**Fig 2:**
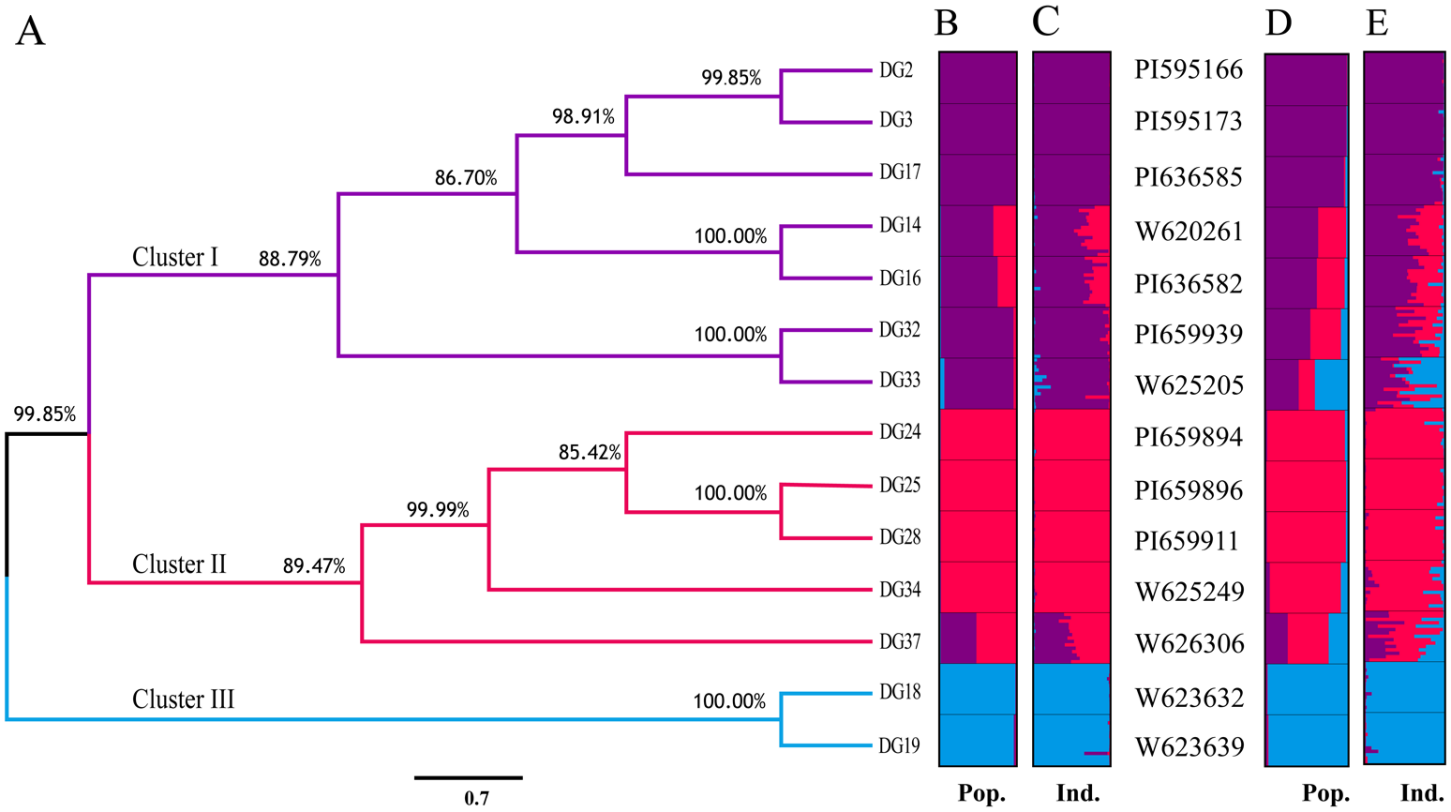
Cluster analysis of *D*. *glomerata*. (A) UPGMA tree of 14 populations. (B) STRUCTURE analysis of 14 populations. (C) STRUCTURE analysis of 210 individuals. (D) STRUCTURE analysis of 14 populations using outlier loci. (E) STRUCTURE analysis of 210 individuals using outlier loci. Three colors represented different potential genetic backgrounds.

Based on binary matrix of AFLP amplification, the UPGMA tree of 14 *D*. *glomerata* populations was constructed, which was composed of three clusters (Fig 2A). The purple cluster (Cluster I), with a bootstrap support value of 88.79%, contained all five populations from Xinjiang (DG2, DG3, DG14, DG16 and DG17) and two populations from Central Asia (DG32 and DG33). The red cluster (Cluster II), with a bootstrap support value of 89.47%, included the five populations from Central Asia (DG24, DG25, DG28, DG34 and DG37). The two populations from Kangding (DG18, DG19) gathered into blue cluster (Cluster III), revealing a highest bootstrap value of 100%. To obtain more robust estimates of population structure, we used a model-based Bayesian clustering method. This STRUCTURE clustering revealed that the LnP(D) was greatest and ΔK reached its maximum when K = 3, demonstrating that all populations fell into three clusters (Fig 2B and 2C). The purple cluster covered DG2, DG3, DG17, DG14, DG16, DG32, DG33 and red cluster covered DG24, DG25, DG28, DG34, DG37, while DG18 and DG19 gathered in the blue cluster, which was entirely identical to the UPGMA tree. The cluster analysis (UPGMA tree and STRUCTURE analysis) suggested that populations or individuals gathered in three clusters that closely matched their geographical distribution. Moreover, the outlier loci identified of AFLP data type was used to detect structure pattern of *D*. *glomerata* using STURCTURE analysis. The results identified three genetic clusters receiving highest support after variance correction (Fig 2D and 2E).

### Principal coordinate analysis and assignment test

**Fig 3:**
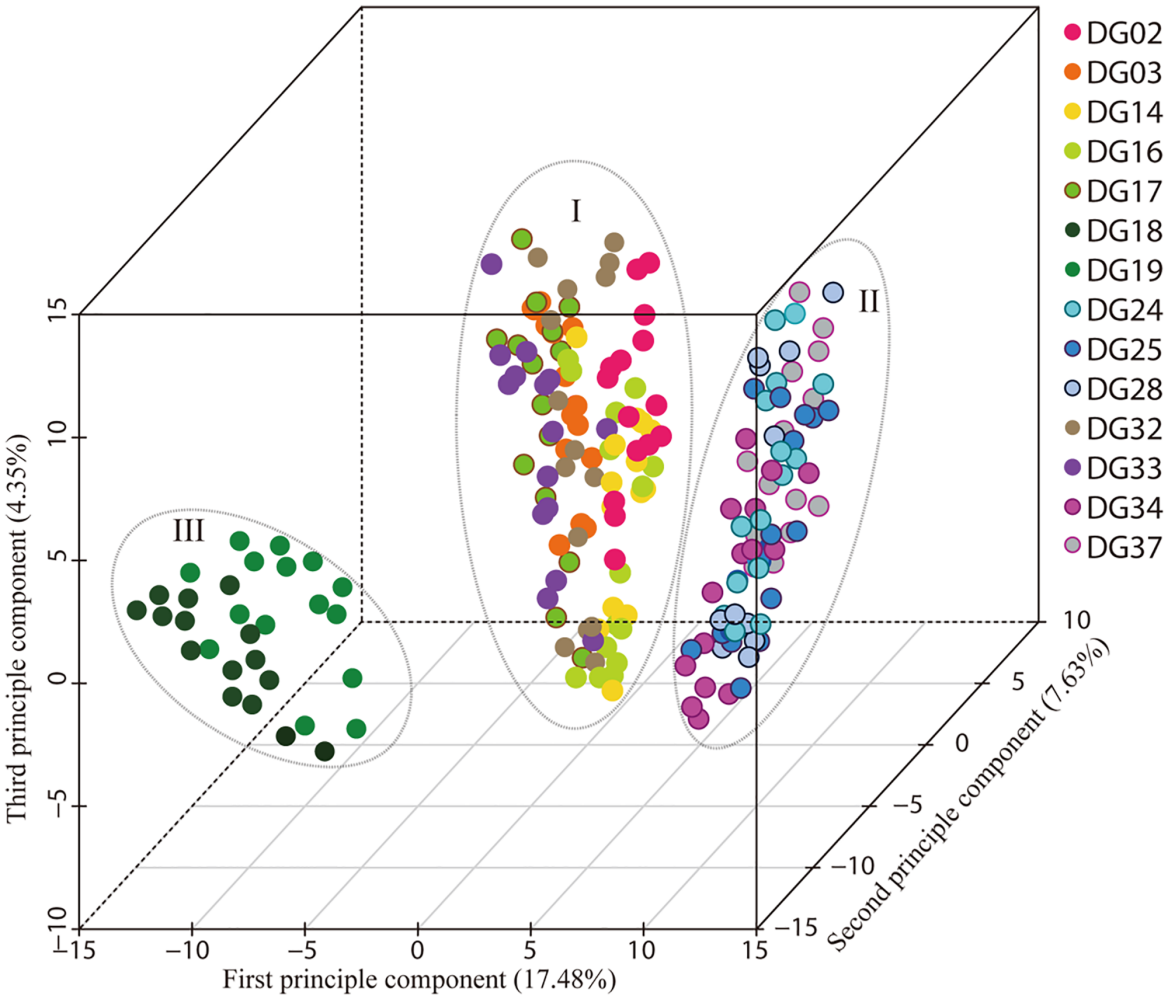
Principle coordinate plot of 210 *D*. *glomerata* individuals. The 15 individuals of per population were represented by the same dots.

The principal coordinate analysis (PCoA) for 210 individuals of *D*. *glomerata* revealed these individuals divided into three groups (Fig 3). All the individuals from Xinjiang gathered into Group I, which also included a few individuals from Central Asia (individuals of DG32 and DG33). The remaining individuals from Central Asia gathered in Group II, and all individuals from Kangding clustered into Group III. Then the first principle vector explained 17.48% of genetic variance, while the second principle vector explained 7.63% and the third principle vector represented 4.35%. The PCoA yielded similar patterns to the two cluster analyses above.

The assignment test indicated that 84.29% of the individuals were allocated back to their original populations with each of these individuals being 100 times more likely that they belonged to this population than any of the other populations (See S3 Fig with article). Population DG3 (100%) showed a highest proportion in assignment test, and population DG33 was the lowest (60%). However, some individuals were not assigned to their original population, *i*.*e*., 5 individuals of population DG33 and 4 individuals of population DG32. There were few individuals that non-assigned to any population.

### Genetic differentiation, gene flow and Mantel test

Analysis of molecular variance (AMOVA) showed that 35.10% of the Nei’s genetic differentiation (*F*_*st*_) occurred among populations whereas larger proportion of variation (64.90%) existed within populations (Table 2). It also suggested that 24.94% of the genetic variance existed among three regions (Table 2). The genetic differentiation between two karyotypes was also calculated in recent regions (CA and XJ), and the result showed that low divergence (*F*_*ct*_ = 0.09) existed between diploid and tetraploid populations of *D*. *glomerata* (Table 2). The Shannon differentiation coefficient (*G’*_*st*_) revealed that 33.69% of the differentiation existed among populations. Mantel test (*r* = 0.7632, *p* < 0.01) revealed a significant isolation-by-distance (IBD) pattern across all sampling sites (Table 3, S4 Fig) As expected, gene flow (*N*_*m*_ = 0.4623) was seriously limited among all populations. According to geographical origins of *D*. *glomerata* germplasm (Central Asia, Xinjiang and Kangding), AMOVA analysis, Mantel test, and gene flow were conducted at smaller scale. The IBD pattern, low *N*_*m*_, high *F*_*st*_ and *G’*_*st*_ were also found between regions (Table 3).

**Table 2:** Analysis of molecular variance of *D*. *glomerata*.

Group	Source of variance	D.f.	Sum of squares	Variance components	Percentage of variance (%)	F-statistic	p-value
Three regions	Among regions	2	2188.235	14.5637	24.93	*F*_*ct*_ = 0.2494	< 0.01
	Among Pops within regions	11	1908.35	9.2602	15.85	*F*_*sc*_ = 0.2112	< 0.01
	Within populations	196	6778.267	34.583	59.21	*F*_*st*_ = 0.4079	< 0.01
	Total	209	10874.852	58.4069			
Two karyotypes	Between karyotypes	1	643.717	5.1712	9.51	*F*_*ct*_ = 0.0951	< 0.01
(CA+XJ)	Between Pops within karyotypes	10	2300.2	12.9169	23.76	*F*_*sc*_ = 0.2376	< 0.01
	Within populations	168	6092.667	36.2659	66.72	*F*_*st*_ = 0.6672	< 0.01
	Total	179	9036.583	54.354			
All populations	Among populations	13	4096.586	18.7026	35.1	*F*_*s*t_ = 0.3510	< 0.01
	Within populations	196	6778.267	34.583	64.9		
	Total	209	10874.852	53.2856			

**Table 3:** List of Mantel test, genetic differentiation and gene flow for *D*. *glomerata* in different regions. Codes: *F*_*st*_, Nei’s genetic differentiation; *G’*_*st*_, Shannon differentiation coefficient; *N*_*m*_, gene flow.

Variable	All populations	KD-XJ	KD-CA	XJ-CA
Mantel test(*r*)	0.7632*	0.8223*	0.8385*	0.4426
*F*_*st*_	0.3510	0.2745	0.2858	0.2108
*G’*_*st*_	0.3369	0.2634	0.2705	0.2066
*N*_*m*_	0.4623	0.6607	0.6247	0.9360

Note:

* = *p* < 0.05.

### Genetic diversity relative to environment

Correlation analyses revealed the population *H*_*j*_ significantly decreased with the increase of geographical altitude (*r* = − 0.7040, *p* < 0.01) (Table 4), which suggested a pattern that low genetic diversity at high elevation. The population *H*_*j*_ weakly and positively correlated to annual precipitation (*r* = − 0.5940, *p* < 0.01), especially for the precipitation in June (*r* = − 0.6971, *p* < 0.01). The population *H*_*o*_ demonstrated a significantly negatively pattern to longitude (*r* = − 0.8176, *p* < 0.01), and weak correlation to latitude (*r* = 0.6842, *p* < 0.01) or annual precipitation (*r* = − 0.6069, *p* < 0.01), but significantly correlated to precipitation in June (*r* = − 0.7017, *p* < 0.01). The population *N*_*p*_ also significantly correlated to latitude (*r* = 0.7278, *p* < 0.01), longitude (*r* = 0.8001, *p* < 0.01) and precipitation in June (*r* = − 0.7174, *p* < 0.01). However, no correlation was found between population LD and environmental factors.

**Table 4:** Pearson correlation analysis between genetic diversity and environmental factors. Codes: *H*_*j*_, Nei’s genetic diversity; *H*_*o*_, Shannon information index; *N*_*p*_, Number of polymorphic loci; LD, linkage disequilibrium.

Variable	Pearson Coefficient	Altitude	Annual Mean Temperature	Annual Precipitation	Temperature in May	Temperature in June	Precipitation in May	Precipitation in June	Latitude	Longitude
*H*_*j*_	*r*	-0.7040	0.3646	-0.5940	0.5580	0.5681	-0.2707	-0.6971	0.5122	-0.4351
	*p*	0.0018	0.0238	0.0073	0.1255	0.0943	0.1782	0.0077	0.0145	0.0207
*H*_*o*_	*r*	-0.3554	0.1386	-0.6069	0.0906	0.5498	-0.4066	-0.7017	0.6842	-0.8176
	*p*	0.0322	0.0251	0.0032	0.2353	0.1187	0.0738	0.0042	0.0035	0.0021
*N*_*p*_	*r*	-0.4085	0.1166	-0.5993	0.1507	0.2366	-0.4873	-0.7174	0.7278	-0.8001
	*p*	0.0273	0.0284	0.0104	0.2116	0.2379	0.0231	0.0034	0.0016	0.0019
LD	*r*	-0.0860	0.0686	-0.1732	0.1649	0.0843	-0.2098	-0.2492	-0.0500	0.3674
	*p*	0.1232	0.1175	0.0904	0.2420	0.1972	0.1341	0.1223	0.2534	0.1634

## Discussion

### AFLP polymorphism and genetic diversity

Population genetic diversity is an important indicator of the capacity for adaptation to varied and changing environment [58]. Analysis of the genetic structure at intraspecific level is important because it is indication of colonization success to new habitats and the capacity for future adaptive change or evolution [59]. Generally, wide-distributed species have much more evolutionary benefits because of their high heterozygosity and low inbreeding depression, thus they could more easily colonize and adapt under changing ecological habitats [60]. Six primer pairs were used for AFLP amplification on 210 individual genomic DNAs of *D*. *glomerata* to detect the genetic diversity and to speculate on the relationships among them. An investigation of genetic diversity of wild *D*. *glomerata* germplasm by using AFLP technique based on bulked sampling method was conducted by Peng et al. [61], which revealed higher PPB (80.50%) and PIC (0.30). The higher PPB and PIC were also reported on *D*. *glomerata* species by Sun et al. (PPB = 97.73%, PIC = 0.2522) [2] and Xie et al. (PPB = 59.29%, PIC = 0.3) [5]. However, a lower PPB of 32% was reported as genetic diversity index for natural *D*. *glomerata* populations by using RAPD and bulked genomic DNAs [62]. The more higher genetic diversity was also revealed by *H*_*j*_ values in previous studies on *D*. *glomerata* in Swiss permanent grassland (*H*_*j*_ = 0.52–0.58) and in three regions in Europe (*H*_*j*_ = 0.44–0.59) [4,63]. The reason for above divergence might be the difference in the source of the germplasm, sampling size, germplasm DNA composition (individual DNA or bulked DNA) and the choice of molecular markers. Overall, the highly genetic variability of *D*. *glomerata* in this study was probably caused by its life traits in long evolutionary histoty, such as outbreeding mating system and efficient pollen dispersal[63].

### Population structure and gene flow among different regions

Gene flow could counteract intraspecific genetic drift and interspecific differentiation, while genetic drift would aggravate interspecific differentiation and decrease species genetic diversity when *N*_*m*_ values are less than one [64,65]. Typically, a high degree of gene flow is very common in self-incompatible and wind-pollinating grass species, leading to low genetic variation among individuals and populations [4,66]. In this study, total *F*_*st*_ = 0.3510 and total *G’*_*st*_ = 0.3369 revealed genetic variance mostly occurred within populations and it was correlated to breeding system of cross-pollination. This pattern of genetic structure was also found by previous studies on *D*. *glomerata* [3,4,62], even on tall fescue [67] and *Lolium multiflorum* [68]. Mantel test suggested a strong and significantly positive correlation between pairwise *F*_*st*_ and geographical distance (*r* = 0.7297, *p* < 0.01), indicating a significant pattern of isolation-by-distance (IBD) across all sampling sites (Table 3). Similar IBD pattern was also found by Sun (*r* = 0.301, *p* < 0.01) [2], Tuna (*r* = 0.64, *p* = 0.01) [62] and Last (*r* = 0.39, *p* < 0.01) [63]. Given the IBD pattern, gene flow was usually limited by long geographical distance, hence the *N*_*m*_ (0.4623) was very low across all sampling sites. Conversely, individuals of neighboring populations would obtain more opportunities of allelic exchange, so they tend to be genetically similar to one another.

Considering the very long geographical distance between Kangding and Xinjiang or Central Asia (more than 1886 km), the expectantly significant IBD patterns were found by Mantel Tests (KD-XJ: *r* = 0.8223, p < 0.01; KD-CA: *r* = 0.8385, p < 0.01) (Table 3). As a result, the gene flow between regions was much limited (KD-XJ: *N*_*m*_ = 0.6607; KD-CA: *N*_*m*_ = 0.6627), and high genetic differentiation (KD-XJ: *F*_*st*_ = 0.2745, *G’*_*st*_ = 0.2634; KD-CA: *F*_*st*_ = 0.2858, *G’*_*st*_ = 0.705) was revealed. Hence, populations of Kangding independently clustered together in cluster analysis. The low gene flow and high differentiation were also detected between Xinjiang and Central Asia (Table 3: *N*_*m*_ = 0.9360, *F*_*st*_ = 0.2108, *G’*_*st*_ = 0.2066), although no significant IBD occurred between them (Table 3. Mantel test: *r* = 0.4426). This structure pattern of *D*. *glomerata* was demonstrated more directly in PCoA that individuals tended to gather in the same group when they had close proximity in geographical distance. This pattern most likely reflects the historical evolutional traits of *D*. *glomerata*. The barrier effects of long geographical distance or high Tianshan Mountains have caused the unidirectional variation of individuals in neutral evolution but led to the directional variation and convergent genotypes of population structure, e.g. genetic drift allowed for populations to evolve traits in their local habitats by fixing or losing of alleles [4,63,69].

Previous revealed that once natural selection has affected the genome of species, outlier loci would show limited differentiation between populations in the case of balancing selection, or extensive differentiation if divergent selection has had an effect [42]. The genetic structure of *D*. *glomerata* also suggested natural selection has participated in population divergence or speciation. For example, outlier levels of genetic differentiation revealed high *F*_*st*_ = 0.4013 and low *N*_*m*_ = 0.3730 across all populations; many populations including DG14, DG16, DG32, D33 and DG37 demonstrated more complex genetic lineage in STRUCTURE analysis (Fig 2D and 2E). It revealed divergent selection has affected ecotypic divergence in the presence of gene flow, which caused local adaption and further accelerated differentiation among populations in different geographical regions or even in a small range of distribution under the long-term selective pressure [37,70].

### Population genetic diversity associated with environmental factors

The correlation between population *H*_*j*_ and geographic altitude was significantly negative (*r* = − 0.7040, p < 0.01), revealing low population genetic diversity in high altitudes. Generally, wind-pollination is an important guarantee for *D*. *Glomerata* to cross fertilize, while this process is easily affected by environmental conditions [1]. Some studies suggested that the success of fertilization process of plant species were reduced in alpine regions due to their adverse environmental conditions, containing intense ultraviolet radiation, strong winds and short growth periods [71]. As a consequence, population sizes gradually decreased, leading to deleterious erosion of genetic diversity by increased inbreeding and genetic draft [72]. Ambient temperature is a considerable factor for growth and development of plants species, especially the low temperature. Conversely, the annual mean temperature, temperature in May and temperature in June did not significantly impact on population *H*_*j*_, *H*_*o*_, *N*_*p*_ or LD in this study (Table 4), because temperature in these sampling sites were not significantly differentiated. Whereas previous studies revealed historical climatic oscillations had potentially influence on contemporary endemic genetic diversity such as the species of glacial refugias in Altay areas and Tianshan Mountains, because migration and expansion generally took place from refugias during interglacial period [73]. For an example, extremely low temperature during glaciation may have caused local extinctions of populations at the top of Tianshan Mountains in Xinjiang, whereas the populations at foot of these mountains may have more favorable climatic conditions. Those alpine populations might derive from the few individuals of lowland populations during interglacial periods, which would then exhibit low genetic diversity and variation [69,74]. Therefore, this is another explanation for the lower genetic diversity in higher altitude.

Pearson analyses revealed *H*_*j*_ was weakly correlated to annual mean precipitation, but significantly related to precipitation in June (Table 4). Typically, facing temperate continental climate, the wet season in Xinjiang and Central Asia always concentrate in every May and June [75], and the two months are the most important flowering period of *D*. *glomerata*. Thus the high rainfall (e.g. continuous heavy rain during flowering stage) may block pollen transmission in some sites. It also partly lead to the decrease in population size and erosion of genetic diversity (Pearson correlation analyses in Table 4: *H*_*j*_ and precipitation in June, *r* = − 0.6971; *H*_*o*_ and precipitation in June, *r* = − 0.7017; *N*_*p*_ and precipitation in June, *r* = − 0.7174). In present study, population *H*_*o*_ and *N*_*p*_ were correlated to latitude or longitude, the similar pattern was also reported on *Stipa grandis* [76] and *Blysmus sinocompressus* [77]. Because the local microenvironment conditions would greatly change (*i*.*e*. ambient temperature, sunlight intensity and duration, wind) in different collection sites, the growth status, evolutionary direction and distributed limit of plant might sharply change and affect the genetic diversity of species.

## Conclusion

In conclusion, the investigation of 14 wild *D*. *glomerata* populations revealed high genetic diversity and high genetic differentiation among populations and regions. The isolation-by-distance pattern was significantly across entire distribution range. As expected, genetic differentiation was high and gene flow was limited not only among all populations but also between adjacent regions. The genetic diversity and structure of *D*. *glomerata* were affected by geographical distance and mountainous isolation. The geographical distribution and genetic relationship of populations were consistent that most likely derived by divergent selection during the long-term neutral and natural evolution. The genetic diversity was also influenced by environment like altitude, precipitation, latitude and longitude.

## Supporting information

S1 Fig Scatter of 352 polymorphic amplified fragment length polymorphisms loci of 210 individuals of Dactylis glomerata in DetSel. *F*_*st*_ (genetic differentiation) is plotted against the log10 of the PO (posterior odds). The vertical line shows the critical PO used for identifying outlier markers. The 32 markers on the right side of the vertical line are candidates for being under positive selection.
(TIFF)

S2 Fig Distribution of *F*_*st*_ values as a function of heterozygosity for interpopulational comparisons. Each dot represent an AFLP locus. The dots in red board are classified as outliers potentially under divergent selection, and the alternative are netural loci.
(TIFF)

S3 Fig Results on individuals assignment conducted in 14 *D*. *glomerata*. populations under minimal log-likelihood difference (MLD = 2). Analysis included 210 individuals of 14 populations and was based on 352 polymorphic AFLP bands. Blue bars represented individuals assigned to their original population; red bars represented individuals assigned to a population different from the original population; brown bars represented individuals not confidently assigned to any of the 14 populations.
(TIFF)

S4 Fig Mantel test between pairwise *F*_*st*_ and geographical distance for14 *D*. *glomerata* populations. (TIFF)

S1 Table List of the 14 wild *D*. *glomerata* populations in this study. Codes: AMT, annual mean temperature; AP, annual precipitation.
(DOCX)

S2 Table Amplification and polymorphism of the PCR products based on AFLP primers. Codes: TNB, total number of band; NPB, number of polymorphic bands; PPB, percentage of polymorphic bands; PIC, polymorphic information content; *H*_*o*_, Shannon information index.
(DOCX)

S1 Text The original binary matrix of 210 individuals. (TXT)
