# Supplementary figures and images for: AFLP-based genetic diversity of wild orchardgrass germplasm collections from Central Asia and Western China, and the relation to environmental factors
Source: PLoS One. 2018 Apr 11;13(4):e0195273. doi: 10.1371/journal.pone.0195273 (PMC5894997; doi:10.1371/journal.pone.0195273)

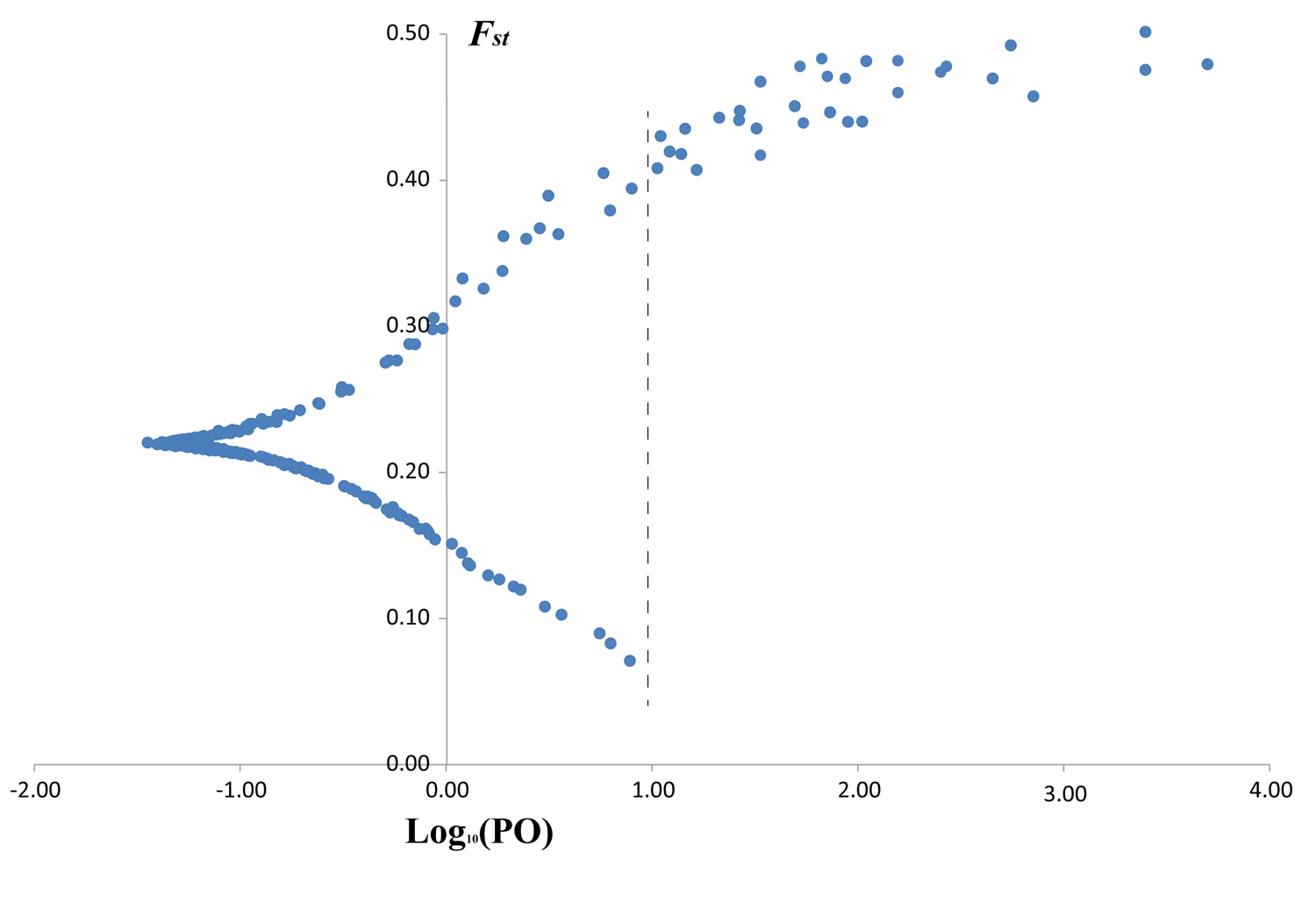

Supplement: S1 Fig — Fst (genetic differentiation) is plotted against the log10 of the PO (posterior odds). The vertical line shows the critical PO used for identifying outlier markers. The 32 markers on the right side of the vertical line are candidates for being under positive selection. (TIFF) [file pone.0195273.s001.tiff]

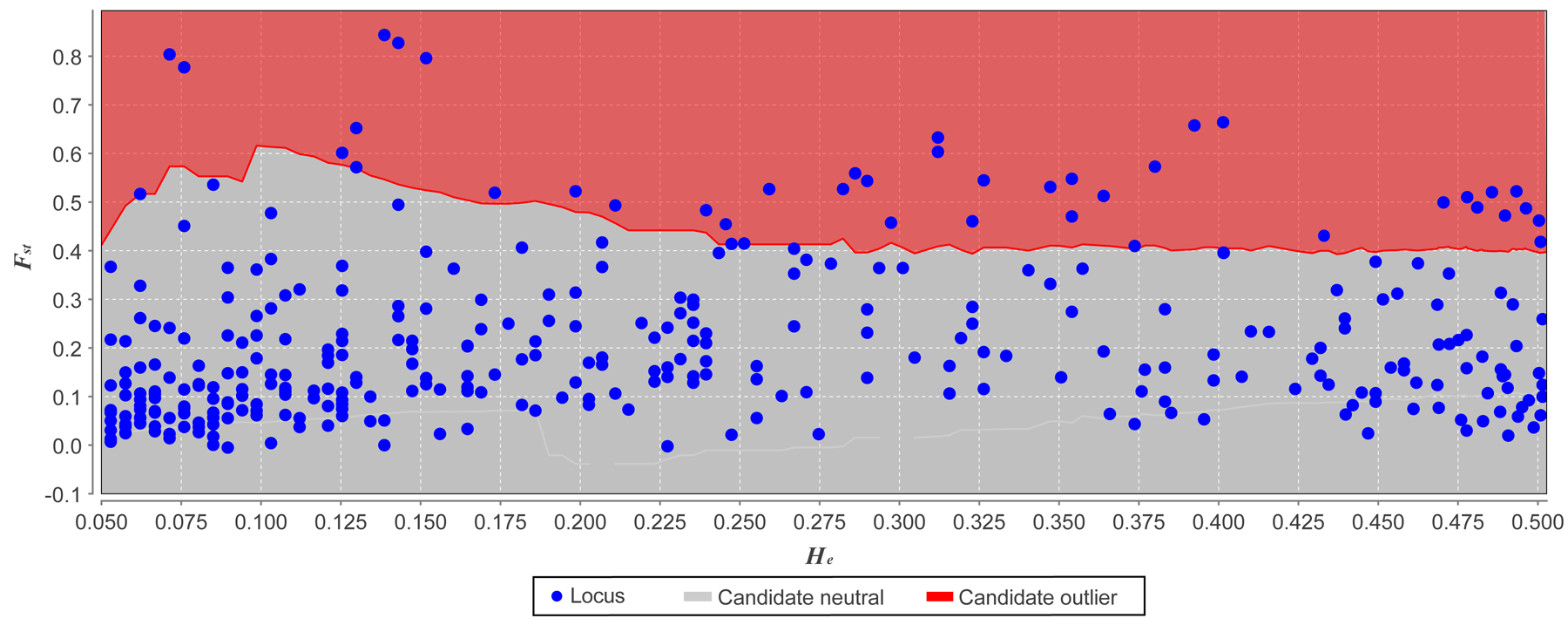

Supplement: S2 Fig — Each dot represent an AFLP locus. The dots in red board are classified as outliers potentially under divergent selection, and the alternative are netural loci. (TIFF) [file pone.0195273.s002.tiff]

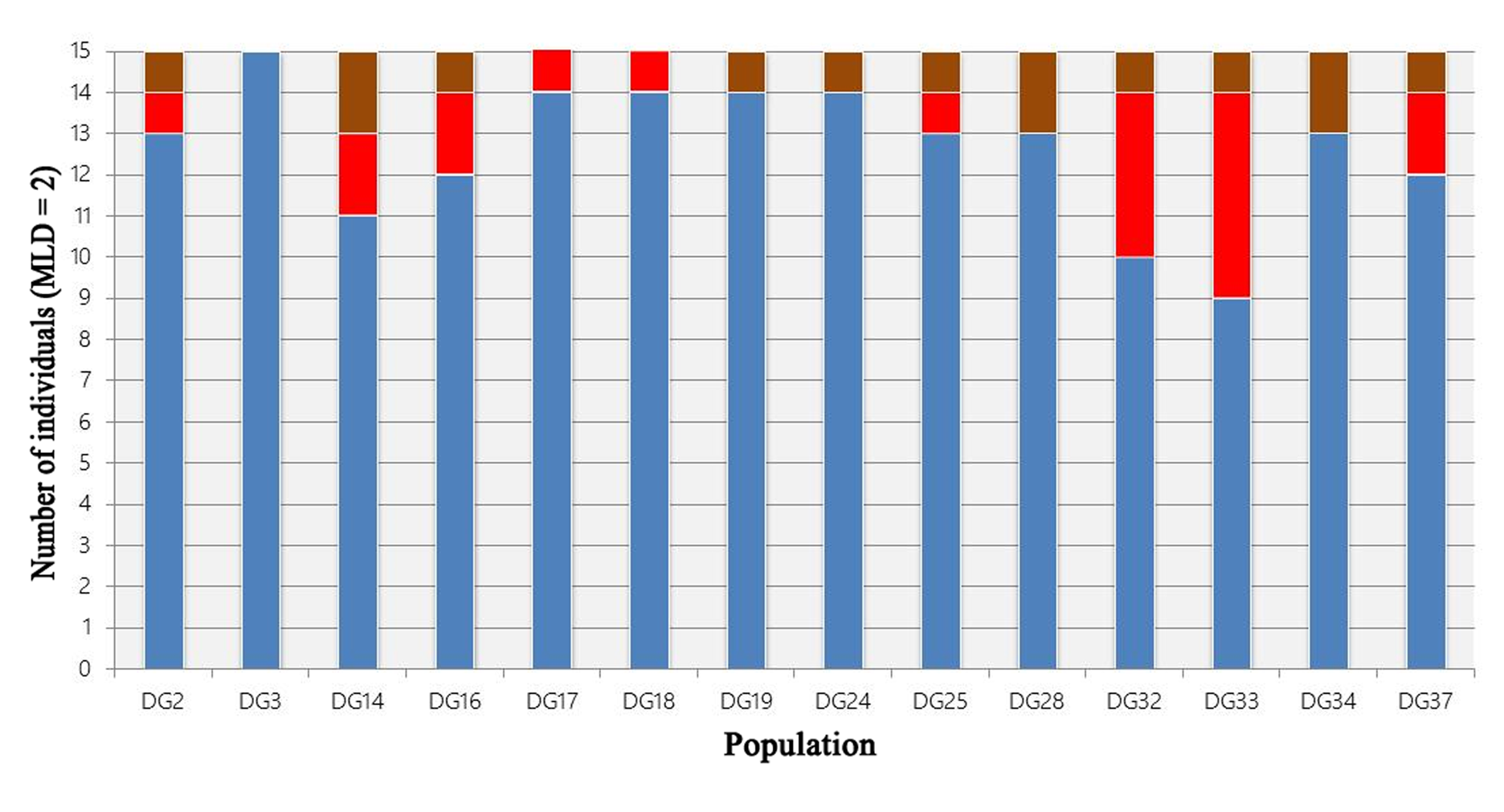

Supplement: S3 Fig — Analysis included 210 individuals of 14 populations and was based on 352 polymorphic AFLP bands. Blue bars represented individuals assigned to their original population; red bars represented individuals assigned to a population different from the original population; brown bars represented individuals not confidently assigned to any of the 14 populations. (TIFF) [file pone.0195273.s003.tiff]

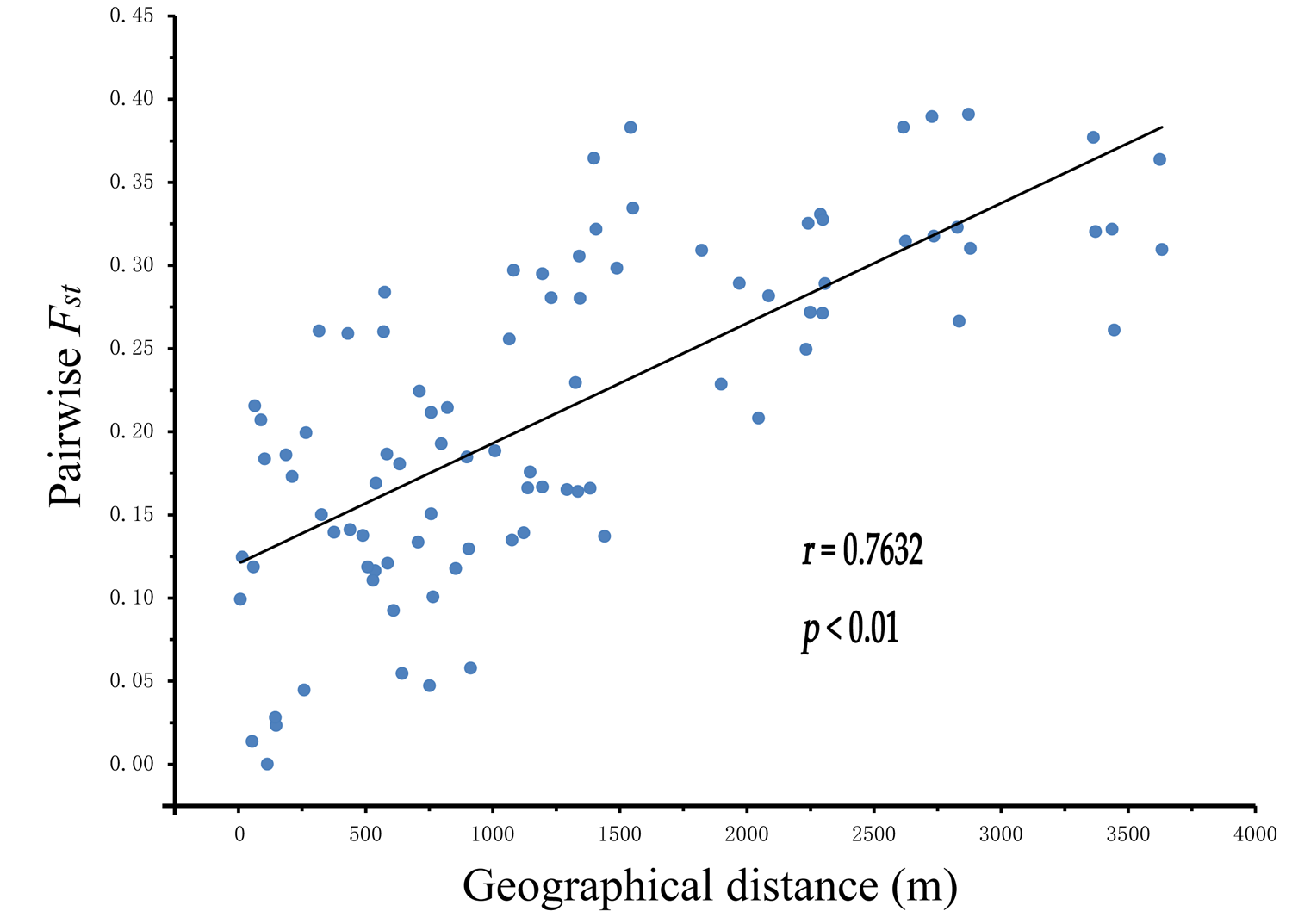

Supplement: S4 Fig — (TIFF) [file pone.0195273.s004.tiff]
