# Supplementary material for: AFLP-based genetic diversity of wild orchardgrass germplasm collections from Central Asia and Western China, and the relation to environmental factors
Source: PLoS One. 2018 Apr 11;13(4):e0195273. doi: 10.1371/journal.pone.0195273 (PMC5894997; doi:10.1371/journal.pone.0195273)
Supplement: S1 Table — Codes: AMT, annual mean temperature; AP, annual precipitation. (DOCX) [file pone.0195273.s005.docx]

| **Name** | **Register ID** | **Ploidy** | **Location** | **Latitude (E)** | **Longitude (N)** | **Altitude (masl)** | **AMT (°C)** | **AP (mm)** | **Habitat** |
| --- | --- | --- | --- | --- | --- | --- | --- | --- | --- |
| DG2 | PI595166 | 4X | Heavenly Lake, Xinjiang, China | 44°7′0″ | 87°58′0″ | 728 | 7.242 | 199 | Uncut, bottom of slope at non-irrigated mountain pasture |
| DG3 | PI595173 | 4X | 44km south of Jimsar, Xinjiang, China | 43°41′0″ | 89°18′0″ | 2105 | 1.183 | 204 | Loam soil, middle pasture, sloping steep hillside pasture |
| DG14 | W620261 | 4X | 40 km east Zhaosu County, Xinjiang, China | 43°9′52″ | 81°37′12″ | 1702 | 4.104 | 367 | Ungrazed meadow that will be cut for hay |
| DG16 | PI636582 | 4X | 45 km northeast Huocheng, Xinjiang, China | 44°13′10″ | 81°9′30″ | 1048 | 6.325 | 306 | Hay meadow next to mountain-fed stream. Clay loam |
| DG17 | PI636585 | 4X | 58 km east of Yili City, Xinjiang, China | 43°27′28″ | 81°5′31″ | 2125 | 1.479 | 489 | Hillside, moderately grazed. Silt loam soil |
| DG18 | W623632 | 4X | 10 km southeast of Kangding, China | 29°59′35″ | 101°53′1″ | 3583 | 4.088 | 930 | Field waste margin, horse bean field. Gravelly loam |
| DG19 | W623639 | 4X | 3 km south of Kangding, China | 29°59′0″ | 101°57′25″ | 3320 | 5.258 | 905 | Field borders. Gravelly loam |
| DG24 | PI659894 | 2X | Bystrovka,Kemin, Kyrgyzstan | 42°48′1″ | 75°55′30″ | 1536 | 5.017 | 248 | Low flood plain terraces in broad valley, gravelly clay loam |
| DG25 | PI659896 | 2X | 20 km east of Cholpon Ata, Kyrgyzstan | 42°39′50″ | 77°13′34″ | 1611 | 5.300 | 314 | Lowest flood plain terrace in broad valley, sandy loam |
| DG28 | PI659911 | 2X | 29 km southwest of Karakol, Kyrgyzstan | 42°19′31″ | 78°14′45″ | 2550 | 1.079 | 344 | Lower canyon, fence-protected pasture, gravelly loam |
| DG32 | PI659939 | 2X | Slope of At-Bashy Range, Kyrgyzstan | 41°14′15″ | 76°21′35″ | 2765 | 0.071 | 245 | Dry bluffs and boggy slopes at native steppe, clay loam |
| DG33 | W625205 | 4X | Khaburobod mountain pass, Tajikistan | 38°40′17″ | 70°44′16″ | 3377 | 0.000 | 796 | Village in Khaburobod mountain pass |
| DG34 | W625249 | 4X | Khorog along the Gunt river valley, Tajikistan | 37°26′26″ | 71°36′30″ | 2642 | 6.263 | 348 | Cultivated small farm fields-cereal and legume crops. |
| DG37 | W626306 | 4X | Dangara towards Nurek, Kulyab, Tajikistan | 38°21′45″ | 69°14′29″ | 1167 | 12.804 | 656 | Roadside in dry mountain grassland |
