# Supplementary material for: AFLP-based genetic diversity of wild orchardgrass germplasm collections from Central Asia and Western China, and the relation to environmental factors
Source: PLoS One. 2018 Apr 11;13(4):e0195273. doi: 10.1371/journal.pone.0195273 (PMC5894997; doi:10.1371/journal.pone.0195273)
Supplement: S2 Table — Codes: TNB, total number of band; NPB, number of polymorphic bands; PPB, percentage of polymorphic bands; PIC, polymorphic information content; Ho, Shannon information index. (DOCX) [file pone.0195273.s006.docx]

| **Primers** | **TNB** | **NPB** | **PPB (%)** | **PIC** | ***H_o_*** |
| --- | --- | --- | --- | --- | --- |
| E42M55 | 99 | 53 | 53.54 | 0.179 | 0.3097 |
| E42M57 | 106 | 60 | 56.60 | 0.198 | 0.2945 |
| E43M85 | 101 | 51 | 50.50 | 0.169 | 0.3107 |
| E85M55 | 109 | 60 | 55.05 | 0.170 | 0.3041 |
| E85M85 | 120 | 63 | 52.50 | 0.165 | 0.3006 |
| E86M85 | 108 | 65 | 60.19 | 0.193 | 0.2961 |
| Total | 643 | 352 | 54.73 | 0.179 | 0.3834 |
| Mean | 107.2 | 58.7 | 54.73 | 0.179 | 0.3026 |
